# An Open Source Low-Cost Wireless Control System for a Forced Circulation Solar Plant

**DOI:** 10.3390/s151127990

**Published:** 2015-11-05

**Authors:** Francesco Salamone, Lorenzo Belussi, Ludovico Danza, Matteo Ghellere, Italo Meroni

**Affiliations:** Construction Technologies Institute, National Research Council of Italy (ITC-CNR), Via Lombardia, 49, 20098 San Giuliano Milanese (MI), Italy; E-Mails: belussi@itc.cnr.it (L.B.); danza@itc.cnr.it (L.D.); ghellere@itc.cnr.it (M.G.); meroni@itc.cnr.it (I.M.)

**Keywords:** DIY, Arduino, low-cost sensor, control system, solar panel, temperature monitoring, Zigbee, Xbee, Bluetooth, WSN, IoT, Open Source hardware, RES

## Abstract

The article describes the design phase, development and practical application of a low-cost control system for a forced circulation solar plant in an outdoor test cell located near Milan. Such a system provides for the use of an electric pump for the circulation of heat transfer fluid connecting the solar thermal panel to the storage tank. The running plant temperatures are the fundamental parameter to evaluate the system performance such as proper operation, and the control and management system has to consider these parameters. A solar energy-powered wireless-based smart object was developed, able to monitor the running temperatures of a solar thermal system and aimed at moving beyond standard monitoring approaches to achieve a low-cost and customizable device, even in terms of installation in different environmental conditions. To this end, two types of communications were used: the first is a low-cost communication based on the ZigBee protocol used for control purposes, so that it can be customized according to specific needs, while the second is based on a Bluetooth protocol used for data display.

## 1. Introduction

There is an ever increasing interest of professionals in the use of shared and customizable hardware solutions. Over the past years several shared projects and low-cost alternative technologies have appeared and grown, allowing end users to approach the electronics in a simple and fast way [1,2,3]. The user becomes supporter and promoter of the “maker” movement and of the Do-It-Yourself (DIY) approach, removing structural and technological obstacles [4]. The spread of this movement has allowed a proliferation of devices always connected in a communicating-actuating network, *i.e.*, a web of objects connected to the network and interconnected to each other named Internet of Things (IoT) [5]. The revolution of DIY is the last in chronological order. After the agricultural and the industrial revolutions, the information age, the so called Third Wave [6], draws upon the read/write functionality of the Internet and digitally-driven design/manufacture, to enable ordinary people to invent, design, make and, sometimes, sell goods and services [7]. Anybody at any location could carry out the principles of DIY philosophy [8,9,10] through enabling technologies, for example Arduino [11] or Genuino [12]. 

In the present article, the DIY approach has been applied to a control system of a forced circulation solar plant using the rapid prototyping Arduino UNO board. In this system the heat transfer fluid is moved using electric pumps and the storage tank is placed inside the building, not close to the solar panel. The device was made using low-cost sensors, available on the market, XBee S2 communication modules, a microcontroller and an *ad-hoc* case realized with a 3D printer. The analyses performed, presented below, have highlighted the good reliability of the sensors compared to professional sensors, which are much more expensive, with a complete data sheet and a calibration report [3]. The XBee modules allow one to overcome the problem related to a cable connection in the case of a great distance between the sensors, making the system more efficient. These modules support a variety of communication protocols, including ZigBee, 802.15.4 and others [13]. The ZigBee network used in this specific contest is pair-type with two nodes, one of which is the coordinator, installed in the internal control station, and the other is the end device which is the core of the external station. The adoption of this protocol offers clear advantages including the possibility to add several nodes and to create new kinds of networks such as, star, mesh and cluster tree. The ZigBee protocol can provide for a mesh-type connection that bridges the distances between two modules, by interposing routers that repeat the signal [14]. As far as the microcontroller is concerned, several manufacturers have proposed quite popular solutions such as Parallax Inc., Coridium Corporation, FTDI, Picaxe, Arduino, as well as many others, all of them inexpensive. Among them, Arduino boards offer one critical advantage: the open source philosophy (both hardware and software), which capitalizes on the massive non-expert community that has grown around the Arduino concept. For the implementation of the case, a 3D printer (PowerWasp) has been applied. This 3D printer implements the fused deposition modeling (FDM) technology and uses polylactide (PLA) for printing. PLA is one of the most eco-friendly 3D printing materials available; it is made from annually renewable resources (corn-starch) and requires less energy to process than traditional (petroleum-based) plastics. Besides 3D printing, PLA is often used in food containers, such as candy wrappers, and biodegradable medical implants, such as sutures.

## 2. Experimental Section 

The solar forced circulation solar plant was built at the Construction Technologies Institute, National Research Council of Italy (ITC-CNR), San Giuliano Milanese, Milan (Latitude 45°24'5" N, Longitude 9°14'58" E). Such a system is widely used in cold areas because the installation of the storage tank inside the building allows the reduction of the heat losses, improving the efficiency of the whole system. The circulation of the fluid is regulated by sensors connected to a management unit that compares the temperature on the top of the panel with that in the storage tank. The external control unit, aimed at measuring the temperature of the panel and at transmitting the data to the management unit, was developed without wiring following the DIY approach, cheaper than the available solution and easier to implement, to avoid the communication problem with the unit inside the building, far away from the solar panel. The system provides for the installation of an external heat exchanger aimed at simulating the hot water consumption or at dissipating the heat when the temperature inside the boiler exceeds a fixed value set as the maximum threshold.

**Figure 1:**
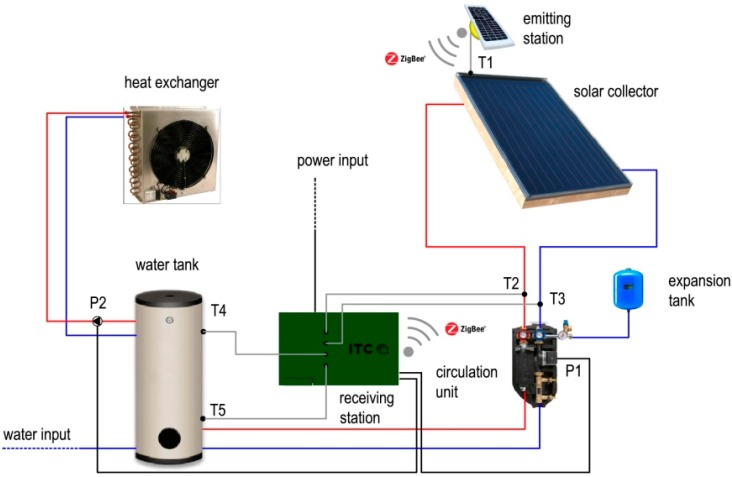
Solar heating system: plant scheme.

The control system of the plant consists of an external wireless and solar energy self-powered unit that transmits the temperature data of the solar panel (T1) to an internal control unit which receives the data and manages the system by controlling the temperature values and the status of the two electric pumps. Figure 1 shows the solar plant scheme pointing to the position of the temperature (Ti-th, expressed in °C) and the pumps management (Pi-th) sensors, in particular:

• T1 is the temperature of the water at the top of the solar panel.
• T2 is the temperature of the outlet water from the panel.
• T3 is the temperature of the inlet water to the panel.
• T4 is the temperature at the top of the storage tank.
• T5 is the temperature at the bottom of the storage tank.
• P1 is the electric pump connecting the solar system to the storage tank.
• P2 is the electric pump connecting the storage tank to the heat exchanger.

### 2.1. External Control Unit

The external wireless control unit, powered by an integrated photovoltaic panel, consists of the following elements:

• Li-ion battery charger module [15].
• 2300 mAh Li-ion battery [15].
• 3 W solar panel [16].
• XBee to USB adapter (alternatively a voltage regulator) [17].
• XBee S2 module [18].
• 10 K thermistor [19].

**Figure 2:**
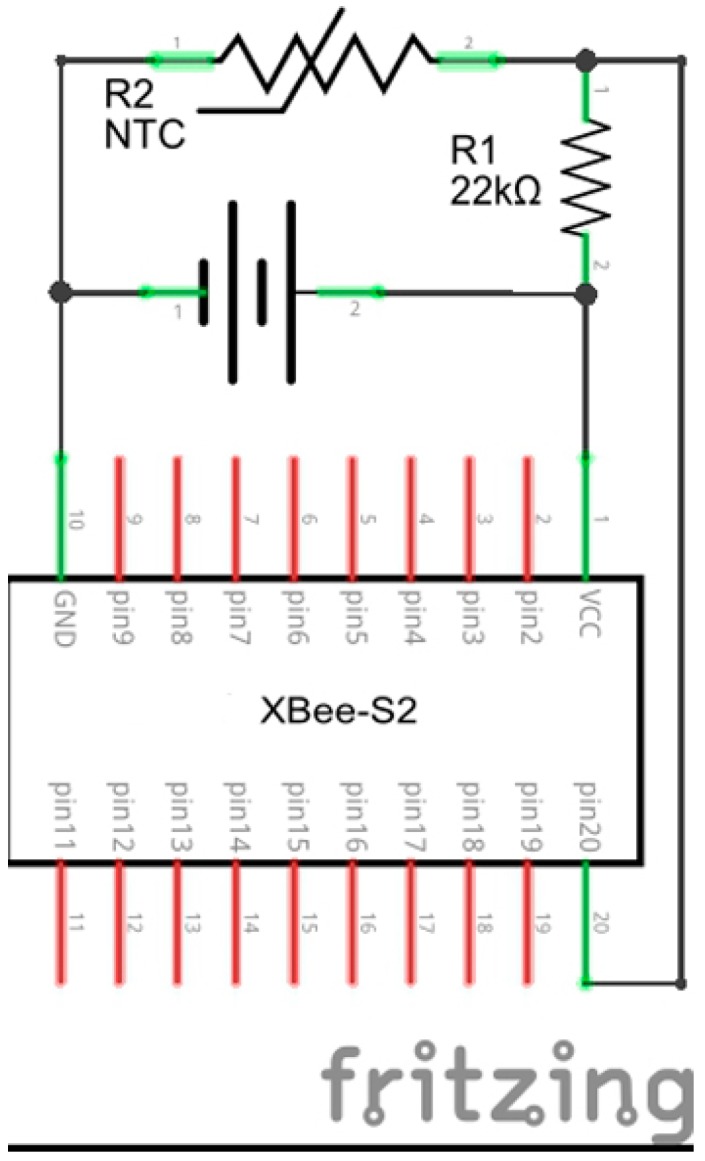
Emitting control unit: connection scheme.

The first four provide continuous feeds to the system. In particular, the Li-ion battery charger module is based on a charger for Li-ion batteries and a DC-DC converter to supply the 5 V. It includes three different inputs: a couple of pin connectors (VCC and GND) for more than 6 V cells and another couple of pin connectors for up to 6 V cells and a mini USB connector. The last two connections go directly to the battery charger, but the first one goes through a voltage regulator whose output is limited to protect the charger from any damage caused by an excessive voltage. This module contains a MAX1555 charger from Maxim to energize the battery: it provides a typical charging current of 280 mA and a voltage of about 4.2 V. The module also contains a MAX1674 chip that converts the 3.7 V in output of the battery into the 5 V. A XBee to USB adapter is used to convert the 5 V in output from the Li-ion Battery charger module into the 3–3.3 V voltage of the XBee module. The XBee to USB adapter is used also to configure the XBee modules using the XCTU Utility provided by DIGI [20]. The 10k thermistor is connected to the XBee S2 by a 21.7 kΩ resistor (Figure 2) exploiting one of the pins that can be configured as analog signals samplers, allowing the potential difference between the single pin and the ground to be read and communicated. The module performs an analog-digital conversion assigning a value between 0 and 1023 to the potential difference, where the maximum value is equal to +1.2 V. Voltage values exceeding the maximum value will always be taken as maximum value. 

**Figure 3:**
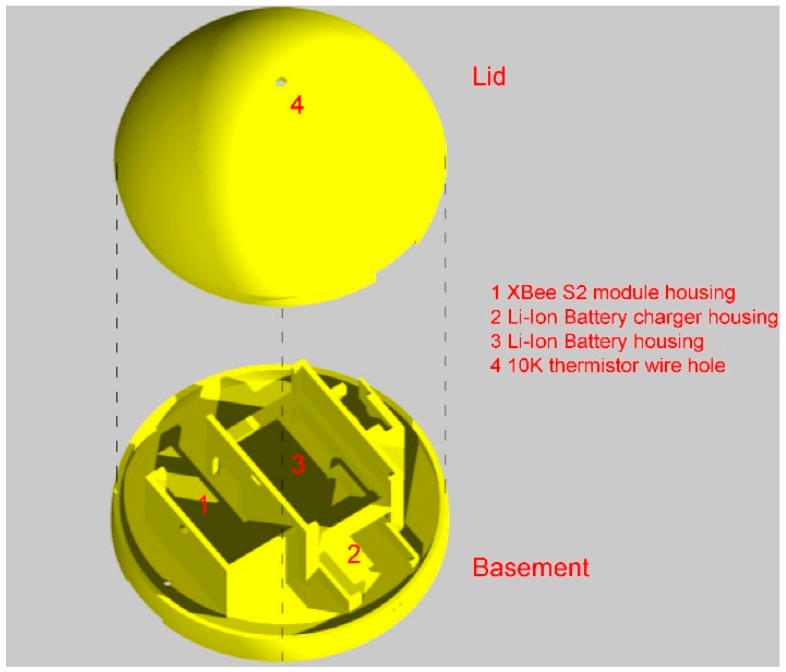
External control unit: assembly scheme rendering.

The sensor detects all the temperature values monitored by the thermistor higher than 20.31 °C, above which the voltage is lower than the maximum value recordable by the module (1.2 V). The external emitting control unit is equipped with a 3D printed hemispherical cap and basement with waterproof interlocking system (Figure 3). 

**Figure 4:**
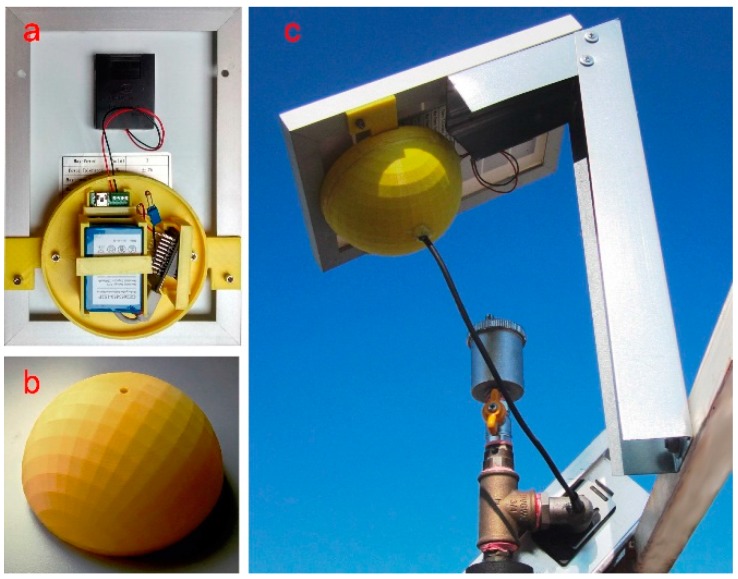
External control unit: (**a**) connection to the photovoltaic pane; (**b**) hemispherical case; (**c**) installation in working conditions.

The circular basement is shaped for housing the electronic components of the unit (Figure 4).

### 2.2. Internal Control Unit

The core of the internal control unit is the open hardware Arduino UNO r3 microcontroller [21]. The following components were used to implement the internal unit:

• Arduino UNO r3 with “sandwich connected” wireless shield [22,23].
• XBee S2 module [18].
• Four DS18B20 waterproof temperature sensors (T2, T3, T4 and T5) [24].
• 2-Channel relay module [25].
• Bluetooth module (optional) [26].
• 3.3 V external module charger (optional, to recharge the Bluetooth module) [27].

**Figure 5:**
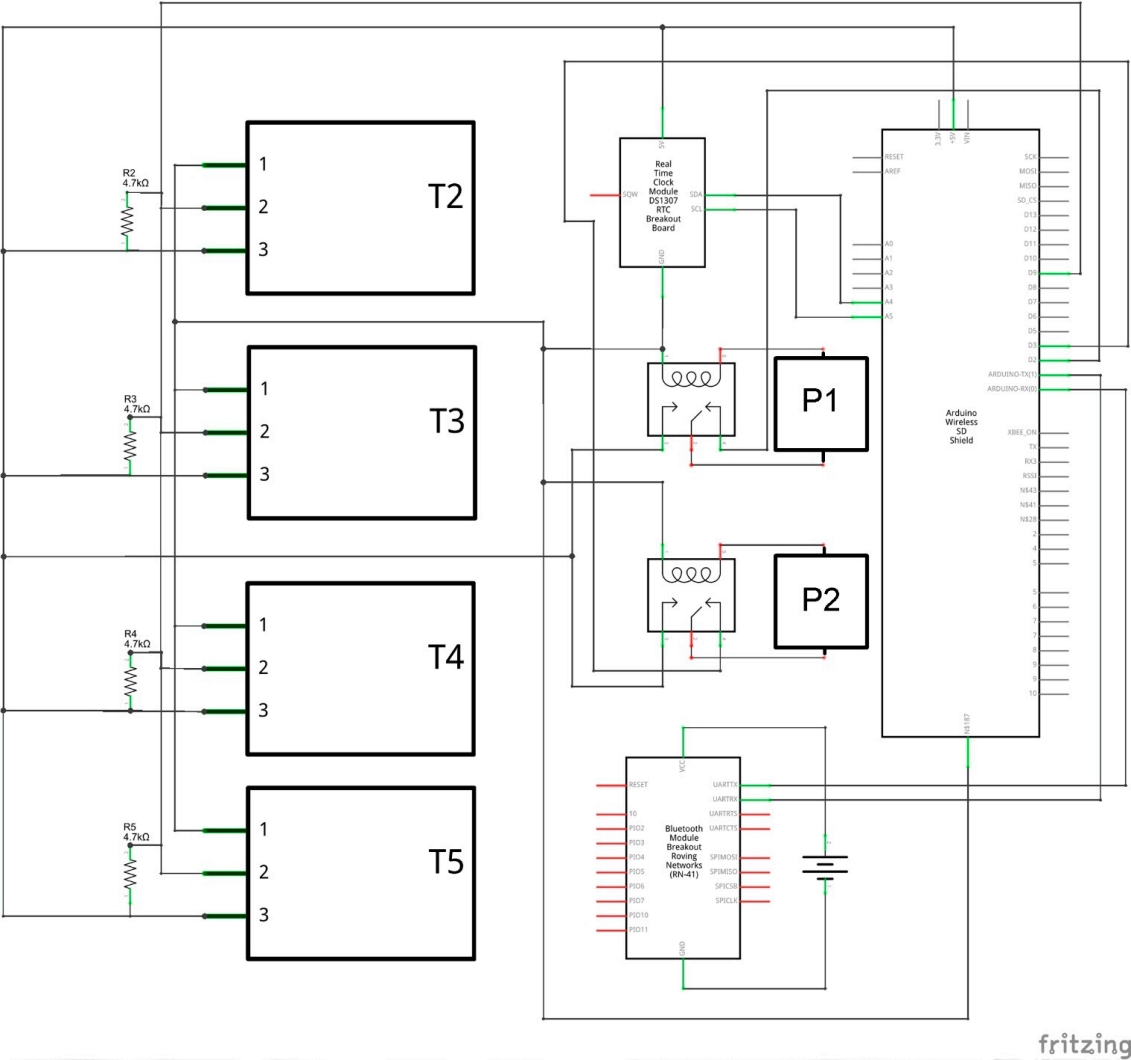
Internal control unit: connection scheme.

**Figure 6:**
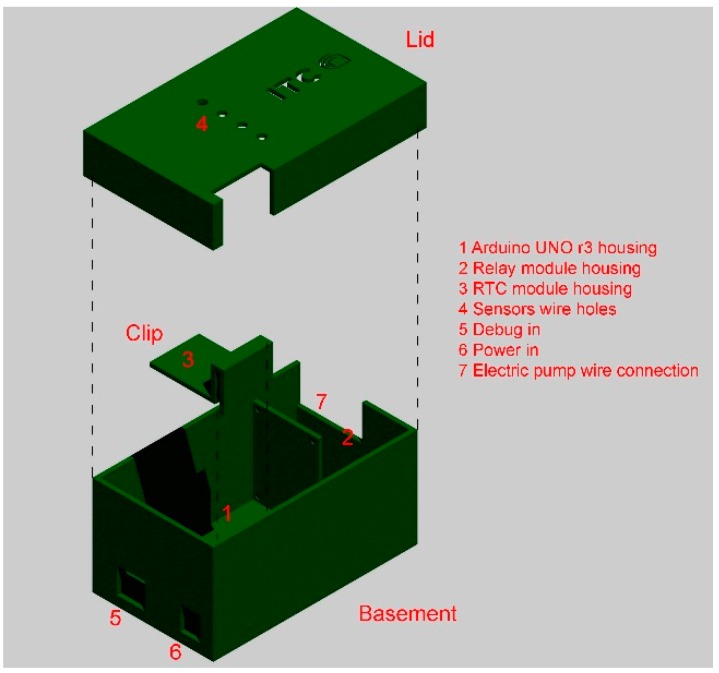
Internal control unit: assembly scheme rendering.

The four waterproof DS18B20 sensors provide 9 to 12-bit (configurable) temperature readings and are connected to pin D9 (Figure 5) because they support 1-Wire interface [28]. The case of the control unit was appropriately designed and realized in PLA using a 3D printer, so that it can be easily assembled and disassembled. It is parallelepiped-shaped and it consists of three elements (Figure 6): a base, a lid and a clip.

The basement contains the slot of the Arduino UNO r3 board and the relay module. The clip element is placed over the base and snap-fitted on one side of the envelope: the element provides the holes for fixing the RTC module. Finally, the lid closes the case providing four central holes for the passage of the cables of the temperature sensors. 

**Figure 7:**
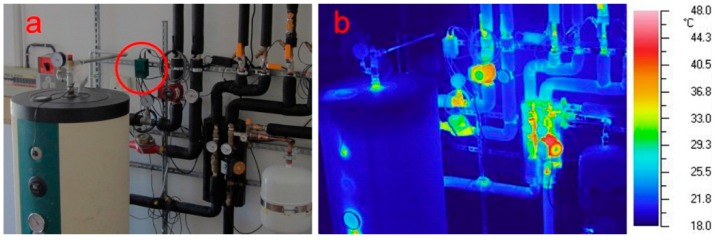
Internal control unit: (**a**) placement in the system; and (**b**) IR thermography.

The control unit is placed inside, close to the pipes that connect the solar panel to the storage tank. The IR thermography performed in different periods allowed to verify that the temperature around the unit was lower than 55–60 °C (Figure 7b), the value at which the PLA changes its shape. The analysis allowed to verify the absence of any hot spots too, both on the mechanical and electronic component of the unit, that might cause problems. 

### 2.3. Data Connection

The overall configuration system provides for the use of two S2 XBee modules (Table 1), that support the ZigBee protocol [29], based on 802.15.4 standard [30]. The XBee module, set as End Device, is the core of the external control unit. The XBee module, set as API coordinator, is connected to the Arduino UNO r3 of the internal receiving control unit through a specific shield.

**Table 1:** XBee modules configuration.

ZigBee End Device AT Configuration	ZigBee API Coordinator Configuration
Address: 0013A20040C281C1	Address: 0013A20040BF97C0
PAN ID 1984	PAN ID 1984
BAUD rate 115200	BAUD rate 115200
D0 ADC [2]	
IR 1388	

According to the description of the MAC frame structure of the ZigBee protocol, the design of communication data frame structure is shown in (Table 2) [31].

**Table 2:** Communication data frame structure.

Sequential Number	Example Value	Purpose
0	7E	Start delimiter
1–2	00–12	Frame length
3	92	Frame type
4–7	00, 13, A2, 00	First part of the sender address
8–11	40, C2, 81, D4	Second part of the sender address
12–13	70, 13	Network address assigned by the coordinator
14	01	Non-broadcast package
15	01	Number of samplings
16–17	00, 00	Bit mask indicating which pins of the XBee module are enabled for digital input
18	01	Bit mask indicating which pins are enabled for analogical input
19–20	03, BC	Each pin enabled for analogical input returns a two-byte reading
21	1C	Checksum

### 2.4. Control Algorithm

**Figure 8:**
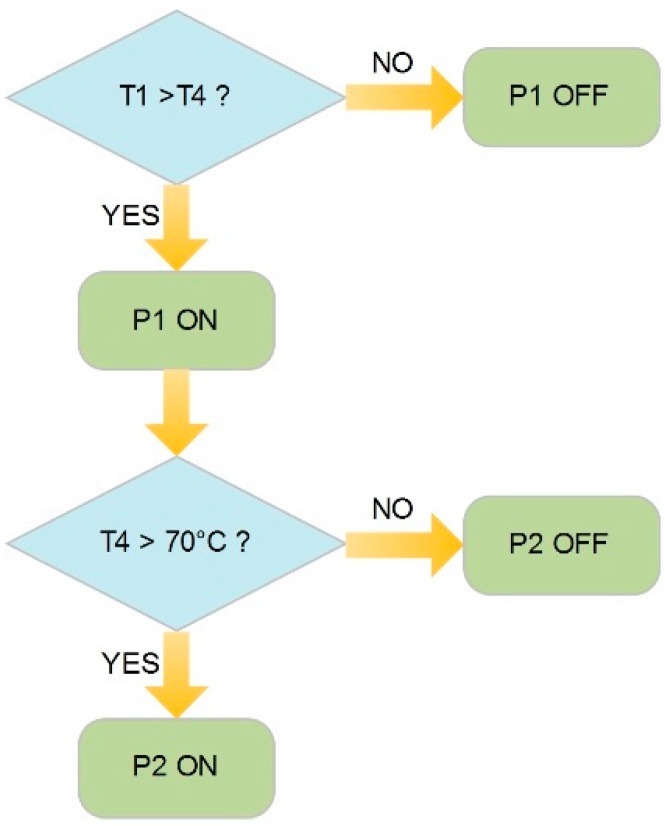
Management of the solar system: flow chart.

The code of the Arduino UNO r3 board allows the control logic shown in Figure 8 to be actuated, comparing the temperature in the solar panel, T1, with that detected at the top of the storage tank, T4. 

The control system turns on the electro pump P1 only if the temperature T1 is higher than T4. If T4 should exceed a threshold value equal to 70 °C the electro pump P2 turns on.

The control algorithm could run with the previous temperature sensors, T1 and T4, applied to the solar panel and the storage tank, as occurs in most commercial systems. For added control and in-depth analysis of the system variables the system was equipped with additional sensors. In particular, the sensor T5, placed at the bottom of the solar tank in addition to T4, allows one to assess the distribution and the possible heat stratification inside the storage system. Sensors T2 and T3 allows one to assess the overall performance of the system, monitoring the inlet and outlet water temperature of the solar panel.

**Figure 9:**
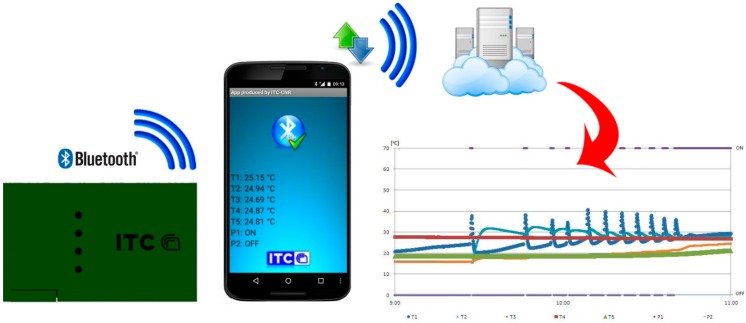
Application scheme for mobile devices (Android OS).

The hardware architecture includes a Bluetooth module for sending data to smartphones: an app for Android devices was made with the aid of MIT App Inventor, a visual programming blocks language for Android OS [32,33]. The control unit, in fact, was designed to record the data on a micro-SD card of the wireless shield, without any display. The app (Figure 9) allows to visualize the temperature data and verify the operating status of the pumping unit on any Android OS device. All data are stored in a cloud server using Wi-Fi or a data connection. 

## 3. Results and Discussion

### 3.1. Sensor Calibration

A calibration activity was conducted to assess the accuracy of the external control unit (T1) and the four DS18B20 sensors (T2–T5). A climate box was used for this purpose, a calibration device able to recreate a controlled temperature and humidity environment, within a range of established values. The box can operate in a temperature range from −40 °C to +180 °C and it can ensure relative humidity values between 10% and 98%. The analysis was conducted by setting a variable temperature profile starting from 15 °C to 85 °C with steps of 10 °C and period of 30 min between the steps.

**Figure 10:**
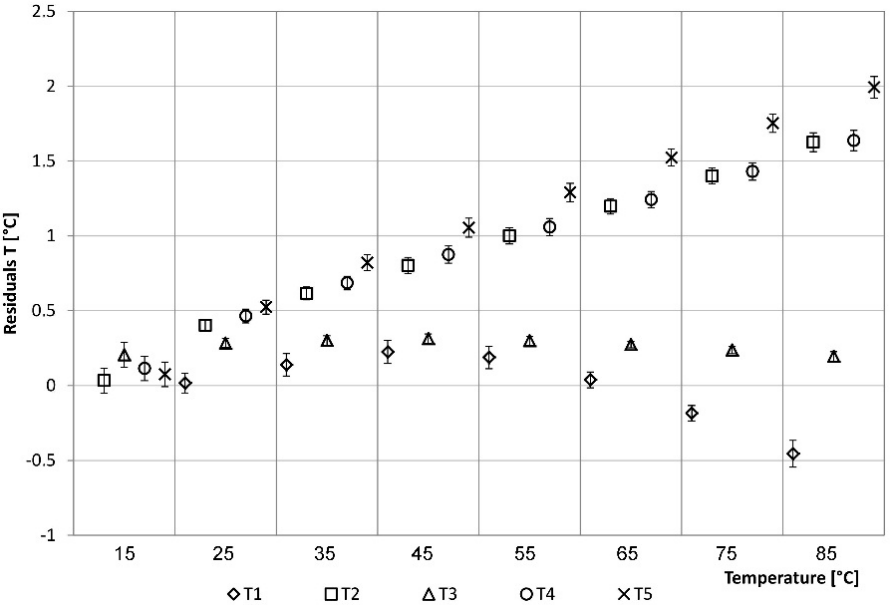
Residuals analysis of the temperatures: verification phase.

Calibration residuals for temperature (°C_Tclimatic_box–°C_Tsensor, i) are considered. The residual analysis shows, in general, lower values detected by the considered sensors than the climatic box settings (Figure 10). In more detail, the T1 sensor detects values that are about 0.1–0.3 °C lower than those of the climatic box, for the range between 35 and 65 °C. Above this range, T1 values are higher than those of the climatic box with a maximum difference of about 0.5 °C. As described above, it should be noted in the graph that T1 sensor detects temperature values higher than 20.31 °C. The values of T3 are constantly lower than those of the climatic box with a difference between 0.3 °C at 45 °C and 0.2 °C at 85 °C. Generally, T1 and T3 sensors provide values that are acceptably accurate for the purposes of the study. 

Sensors T2, T4 and T5 tend to underestimate the real temperature value as the temperature increases, up to a difference of about 1.5–2 °C corresponding to the 85 °C set-point. 

**Figure 11:**
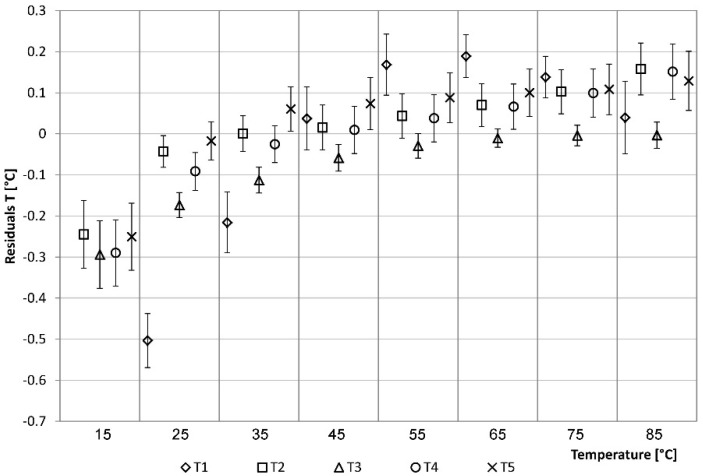
Residuals analysis of the temperatures: correction phase.

The data of sensors T2, T4 and T5 deviate from the values detected by the climatic chamber, showing an unacceptable lack of accuracy. A correction of the trend of temperatures for the different ranges was necessary for use in the monitoring system. For each sensor, the calibration curve (linear equation) was calculated as a function of the values recorded by the climatic chamber. The equations were included within the code of the Arduino board mounted inside the control and management unit box. As a result of the correction, it can be noted that the sensors provide good accuracy over the overall range, correcting in particular the divergent behavior with increasing temperature (Figure 11).

### 3.2. Application to the Real Plant

**Figure 12:**
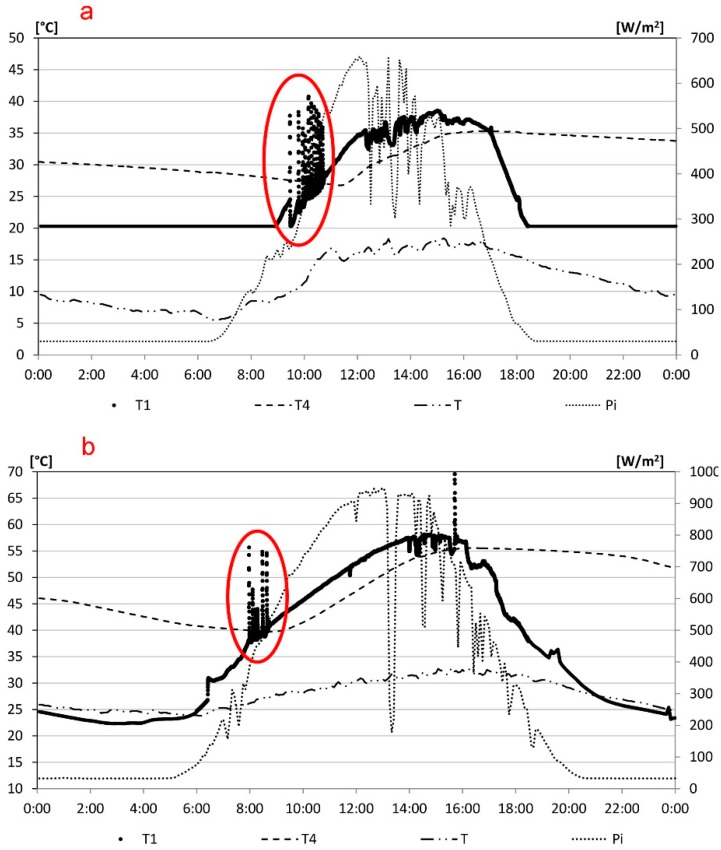
Temperature trend of the sensors T1 and T4: (**a**) 19 March 2015; (**b**) 9 July 2015.

The realized and properly calibrated device was applied to the solar thermal plant, described above. The experimental campaign was conducted both in winter and summer. Results obtained on 19 March and 9 July 2015, are shown, chosen as reference days. Table 3 and Figure 12 show the maximum and minimum temperature and the peak of solar radiation and the trend of the outside temperature, T, the incident solar radiation on the horizontal plane, Pi, and the temperatures detected by the sensors T1 and T4 in the two days, respectively. In particular, the trend of T1 in the morning hours is affected by the control algorithm which causes rapid on/off switching sequences. 

**Figure 13:**
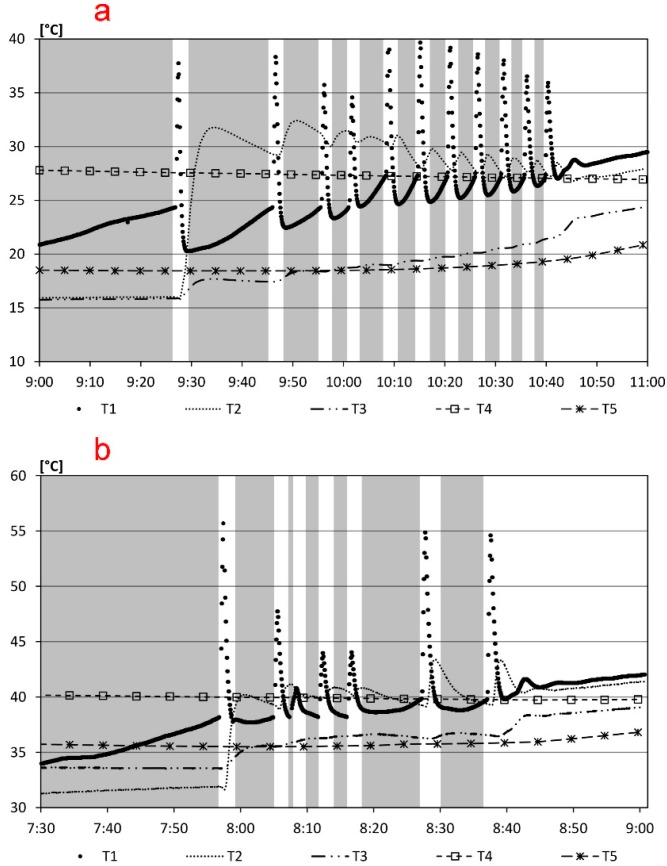
Details of the temperatures detected by the sensors T1–T5: (**a**) 19 March 2015, 9:00 ÷ 11:00; (**b**) 9 July 2015, 7:30 ÷ 9:00. In grey period of P1 switched off, in white period of P1 switched on.

The alternation of the bands, grey and white, of Figure 13 points out the switching off and switching on period of the electric pump P1, respectively, due to the control algorithm. The temperature at the top of the solar panel, T1, increases due to solar radiation. When T1 exceeds T4 the electric pump P1 switches on and the water flows in the panel and in the storage tank with the decrease of T1. By increasing external temperature and solar radiation, the switching on and off steps are more frequent until T1 is higher than T4 and the electric pump is always turned on. On 9 July performances observed from 07.30 to 09.00 are similar, although with fewer on/off cycles and with higher temperatures than 19 March. 

**Table 3:** Daily climate data of the reference days.

	19 March 2015	9 July 2015
**T_min_ (°C)**	5.88	23.80
**T_max_ (°C)**	17.52	32.80
**P_i_ (W/m^2^)**	600	950

Taking into account the detected outside temperature and the incident solar radiation on a horizontal plane, it is possible to calculate the yield of the solar thermal system, ŋ. This value is useful because it allows the proper functioning of the system to be assessed. In this specific case starting from the value of the incident solar radiation on the horizontal plane the incident radiation on the solar panel (P_i_) was determined, calculated considering the tilt and orientation of the solar panel, the latitude and longitude of the location and the albedo [34]. The datasheet of the solar panel contains the data necessary to determine the yield curve given by the following formula:

(1) $$\text{η}={\text{ η}}_{0}-\;\frac{K_{1}∆\text{T}}{P_{i}}\;-\;\frac{K_{2}∆{\text{T}}^{2}}{P_{i}}\;\left[\text{\%}\right]$$

with:

(2) $$∆\text{T}=\frac{\left(\text{T}2+\text{T}3\right)}{2}\;–\text{ T}\;\left[\text{K}\right]$$

where:

ŋ_0_ is equal to 74.0 (%) (from datasheet);
k_1_ is equal to 3.89 (W/m^2^·K) (from datasheet);
k_2_ is equal to 0.018 (W/m^2^·K^2^) (from datasheet);
P_i_ is the incident solar radiation (W/m^2^).
T is the external temperature (°C).
T2 is the temperature of the outlet water from the panel (°C).
T3 is the temperature of the inlet water to the panel (°C).

Table 4 shows the values of the calculated yields as a function of the measured data in some hours of 19 March and 9 July 2015. The yield values during running hours of the solar system is between 73% and 74%, indicating a good behavior of the system.

**Table 4:** Efficiency of the solar panel.

Time	P_i_ (W/m^2^)	∆T (K)	ŋ (%)
08:00 19 March 2015	114.31	8.53	73.70
12:00 19 March 2015	776.59	12.38	73.93
16:00 19 March 2015	452.62	17.12	73.84
20:00 19 March 2015	26.48	6.55	73.01
08:00 9 July 2015	207.41	6.70	73.87
12:00 9 July 2015	879.03	17.67	73.92
16:00 9 July 2015	631.65	21.84	73.85
20:00 9 July 2015	39.92	8.47	73.14

## 4. Conclusions and Future Work

The system, implemented following the DIY philosophy and the use of open hardware and low-cost sensors, allows a solar thermal system with forced circulation to be independently managed, using temperature data from a self-powered wireless station. This system represents an extremely competitive and flexible solution compared to the commercial systems currently available on the market. The analyses conducted so far demonstrate how it is possible to optimally manage a solar system, reducing costs, with the use of appropriately calibrated low-cost sensors. The architecture based on Arduino and a 3D printed case makes it easy to replicate, to use and to customize it. Using a wireless and solar-powered outdoor station instead of a temperature sensor connected by wire allows for a simplified installation and also for an optimized management.

Future developments of the system, with a view to the DIY approach and cost reduction, provide for the possibility to install a low-cost pyranometer that can detect the effective incident solar radiation, in order to determine the actual yield of the solar system. Researchers at the Polytechnic School of the University of Huelva (Spain) have reported a new photodiode-based pyranometer [2] made using self-construction techniques and low-cost sensor. This tool could be connected to the external control unit with the same tilt and orientation of the solar panel or be provided with an XBee module of its own for a direct connection with the internal control unit. In this way, the control unit may individually calculate the performance of the system giving alerts to the users through the mobile device.
